# *Drosophila* caspase activity is required independently of apoptosis to produce active TNF/Eiger during nociceptive sensitization

**DOI:** 10.1038/cddis.2016.474

**Published:** 2017-05-11

**Authors:** Juyeon Jo, Seol Hee Im, Daniel T Babcock, Srividya C Iyer, Felona Gunawan, Daniel N Cox, Michael J Galko

**Affiliations:** 1 Department of Genetics, University of Texas MD Anderson Cancer Center, Houston, TX, USA; 2 Genes and Development Graduate Program, Graduate School of Biomedical Sciences, University of Texas MD Anderson Cancer Center, Houston, TX, USA; 3 Neuroscience Graduate Program, Graduate School of Biomedical Sciences, University of Texas MD Anderson Cancer Center, Houston, TX, USA; 4 Neuroscience Institute, Georgia State University, Atlanta, GA, USA; 5 Department of Biochemistry and Cell Biology, Rice University, Houston, TX, USA

## Abstract

Tumor necrosis factor (TNF) signaling is required for inflammatory nociceptive (pain) sensitization in *Drosophila* and vertebrates. Nociceptive sensitization in *Drosophila* larvae following UV-induced tissue damage is accompanied by epidermal apoptosis and requires epidermal-derived TNF/Eiger and the initiator caspase, Dronc. Major gaps remain regarding TNF function in sensitization, including the relationship between apoptosis/tissue damage and TNF production, the downstream signaling in this context, and the target genes that modulate nociceptive behaviors. Here, apoptotic cell death and thermal nociceptive sensitization are genetically and procedurally separable in a *Drosophila* model of UV-induced nociceptive sensitization. Activation of epidermal Dronc induces TNF-dependent but effector caspase-independent nociceptive sensitization in the absence of UV. In addition, knockdown of Dronc attenuated nociceptive sensitization induced by full-length TNF/Eiger but not by a constitutively soluble form. UV irradiation induced TNF production in both *in vitro* and *in vivo*, but TNF secretion into hemolymph was not sufficient to induce thermal nociceptive sensitization. Downstream mediators of TNF-induced sensitization included two TNF receptor-associated factors, a p38 kinase, and the transcription factor nuclear factor kappa B. Finally, sensory neuron-specific microarray analysis revealed downstream TNF target genes induced during thermal nociceptive sensitization. One of these, *enhancer of zeste* (*E(z)*), functions downstream of TNF during thermal nociceptive sensitization. Our findings suggest that an initiator caspase is involved in TNF processing/secretion during nociceptive sensitization, and that TNF activation leads to a specific downstream signaling cascade and gene transcription required for sensitization. These findings have implications for both the evolution of inflammatory caspase function following tissue damage signals and the action of TNF during sensitization in vertebrates.

Nociceptive sensory neurons often sensitize following inflammation or tissue damage.^1^ These changes foster protective withdrawal behaviors during healing.^2^ *Drosophila* larvae are a powerful model for nociceptive biology because they have a relatively simple peripheral nervous system^3, 4^ and conserved genes that underlie neuronal architecture, synaptic transmission, and nociceptive processing.^5^ *Drosophila* larvae exhibit an aversive, corkscrew-like rolling response distinct from normal locomotion when exposed to noxious thermal or mechanical stimuli.^6^ This nociceptive behavior can be precisely quantified across a range of temperatures.^7^

During *Drosophila* UV-induced nociceptive sensitization, the cytokine TNF/Eiger^8^ is produced by apoptotic epidermal cells. It acts through its receptor, TNFR/Wengen, in nociceptive sensory neurons to mediate thermal allodynia – aversive withdrawal to previously innocuous temperatures. This role of TNF/TNFR is phylogenetically conserved, as they mediate various types of sensitization in vertebrates.^9, 10, 11, 12^ TNF/Eiger is not required for larval epidermal apoptosis,^13^ as it is in *Drosophila* photoreceptors and in some vertebrate tissues.^14, 15^ As TNF/Eiger originates from dying epidermal cells yet acts through its receptor in nociceptive sensory neurons, substantial questions remain regarding how this transmembrane ligand is activated to mediate sensitization.

Although TNF/Eiger is dispensable for UV-induced epidermal apoptosis, the initiator caspase Dronc is required within the epidermis for both epidermal apoptosis and thermal allodynia development.^13^ Dronc is the initiator caspase for apoptosis in most fly tissues.^16^ In the absence of an apoptosis-inducing signal, the death-associated inhibitor of apoptosis protein 1 (DIAP1) prevents the activation of Dronc.^17^ This inhibition is released by the expression of pro-apoptotic genes such as *reaper*, *hid*, and *grim.*^18^ Dronc interacts with death-associated APAF-1-related killer (Dark) to form an apoptosome that activates effector caspases such as Drice and Dcp-1.^19^ These cleave the cellular substrates that lead to apoptosis.^20, 21^ In addition to their roles in apoptosis, *Drosophila* caspases also function non-apoptotically in sperm individualization,^22^ compensatory proliferation,^23^ and innate immunity.^24^

The binding of TNF to TNFR recruits adapters such as TNF receptor-associated factors (TRAFs) to transduce signals. This binding ultimately activates nuclear factor kappa B (NF-*κ*B).^25^ Although the mechanism of NF-*κ*B activation varies by cell type, assembly of downstream mediators at TNFR generally recruits the I*κ*B kinase, resulting in the phosphorylation and degradation of NF-*κ*B inhibitor (I*κ*B).^26^ Free NF-*κ*B then translocates to the nucleus and activates target gene expression. Caspases and mitogen-activated protein kinases (MAPKs) can also be targeted by TNF signaling and can lead to proliferation, cell death, and inflammation.^27, 28^ TNF signaling and NF-*κ*B are associated with both acute and chronic pain.^29, 30, 31, 32, 33, 34, 35^ The transcriptional consequences of TNFR signaling and NF-*κ*B activation in damage-sensitized nociceptive sensory neurons, however, remain unclear.

Here we show that the initiator caspase, Dronc, is required for both apoptosis and generation of an active TNF signal in irradiated epidermal cells. These activities are genetically and procedurally separable. The allodynia-promoting function of Dronc requires TNF but not downstream caspases. Indeed, when TNF is expressed ectopically in nociceptive sensory neurons, an unexpected requirement for Dronc is revealed. We have identified multiple conserved downstream signaling factors and a novel target gene that are required for nociceptive sensitization following TNF-mediated activation of sensory neurons. The epigenetic factor *enhancer of zeste (E(z))*, which is upregulated by TNF signaling in nociceptive sensory neurons, is also required for sensitization. Together, these data reveal the full TNF signaling architecture, from ligand activation/production to signal transduction to downstream target gene activation, that is necessary for nociceptive sensitization following tissue injury.

## Results

### UV-induced cell death and thermal allodynia are genetically separable

**Figure 1:**
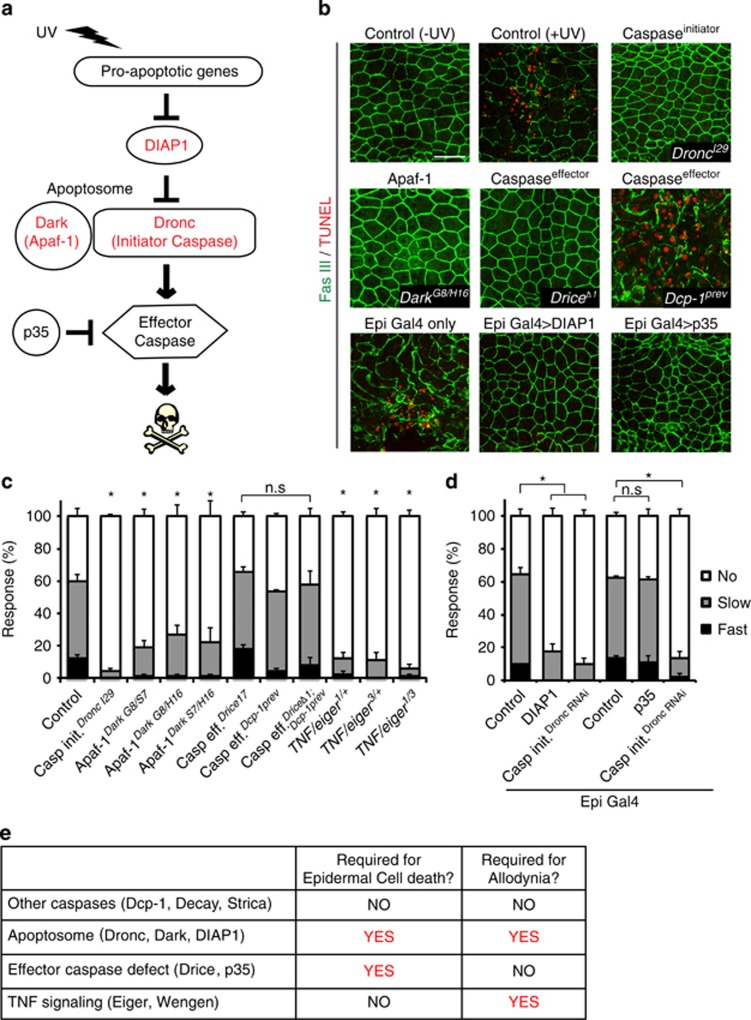
Genetic separation of cell death and thermal allodynia. (**a**) Schematic of canonical cell death pathway in *Drosophila*. (**b**) Larval epidermal staining 24 h after UV irradiation. Anti-Fasciclin-3 antibody (membranes, green) and TUNEL labeling (apoptotic cells, red) were used. Bar, 50 *μ*m (**c** and **d**) Measurement of UV-induced thermal allodynia at 38 °C, 24 h after UV irradiation. Larval behavior was categorized as ‘no withdrawal’ (white), ‘slow withdrawal’ (gray, response between 6 and 20 s), or ‘fast withdrawal’ (black, response≤5 s) in this and other figures *n*=3 sets of 30 larvae, error bars represent S.E.M, and *P<0.05 for this and all subsequent figures. (**e**) Table of relevant genotypes that show or do not show epidermal cell death and UV-induced thermal allodynia

To investigate whether components of the canonical cell death pathway (Figure 1a) are involved in UV-induced apoptosis and thermal allodynia, we first examined morphological damage in larvae mutant for apoptotic genes. The irradiated epidermis of control larvae exhibited disrupted epidermal cell morphology and terminal deoxynucleotide transferase dUTP nick end labeling (TUNEL) staining in their dorsal epidermis *versus* unirradiated controls (Figure 1b). By contrast, mutations in *Dronc*,^16^ *Dark*,^36^ and *Drice*^37^ prevented both morphological disruption and TUNEL staining (Figure 1b). The *Dcp-1* single mutant^38^ failed to block UV-induced apoptosis, suggesting that *Drice* may be the main epidermal effector caspase (Figure 1b). The full apoptosome, defined as the complex of the initiator caspase Dronc and the Apaf-1 adapter protein Dark, was required for apoptosis and allodynia. Consistent with previous tissue-specific knockdowns,^13^ homozygous null mutants for *Dronc* failed to exhibit morphologic hallmarks of apoptosis or thermal allodynia (Figures 1b and c). Three different transheterozygous combinations of *Dark* mutant alleles also failed to undergo epidermal apoptosis (Figure 1b; Supplementary Figure 1) and to induce thermal allodynia (Figure 1c), suggesting that apoptosome formation is required for both processes. However, when we looked further downstream at null mutants for effector caspases, *Drice* mutants, which block epidermal cell death (Figure 1b), still showed strong thermal allodynia (Figure 1c). This was surprising because Drice is a well-known downstream target of Dronc in the context of cell death.^39, 40^ Although *Dcp-1* single mutants did not block UV-induced epidermal apoptosis, we examined the possibility that *Drice* and *Dcp-1* might cooperate to induce thermal allodynia. *Drice* and *Dcp-1* double mutants still developed thermal allodynia, however, despite a complete block of epidermal apoptosis (Figures 1b and c).

The results presented above predict that inhibiting the apoptosome should block both cell death and thermal allodynia, whereas inhibiting effector caspases should block only cell death. To test this hypothesis, we expressed transgenes that inhibit either Dronc (*UAS-DIAP1*) or effector caspase (*UAS-p35*) activity^41^ using the epidermal-specific *e22C-Gal4* driver.^42^ Overexpression of either transgene blocked cell death (Figure 1b). Thermal allodynia upon overexpression of p35 did not differ from Gal4 or UAS-alone controls (Supplementary Figure 2), whereas overexpression of DIAP1 inhibited the development of thermal allodynia (Figure 1d), suggesting that only Dronc, not Drice or Dcp-1, is required to induce thermal allodynia. There were no defects in baseline thermal nociception (absence of tissue injury) when apoptosome or effector caspase function was lacking (Supplementary Figures 1 and 2). Overall, these results demonstrate that Dronc has a non-apoptotic function in promoting thermal allodynia, which must be exerted independently of the effector caspase Drice. Interestingly, in confirming previous results that TNF/Eiger is required for thermal allodynia, we noticed that *eiger* mutants are haploinsufficient for thermal allodynia (Figure 1c). Taken together, our findings indicate that apoptosis and thermal allodynia are completely genetically separable (Figure 1e).

### UV-induced thermal allodynia can be evoked below the threshold for UV-induced apoptosis

**Figure 2:**
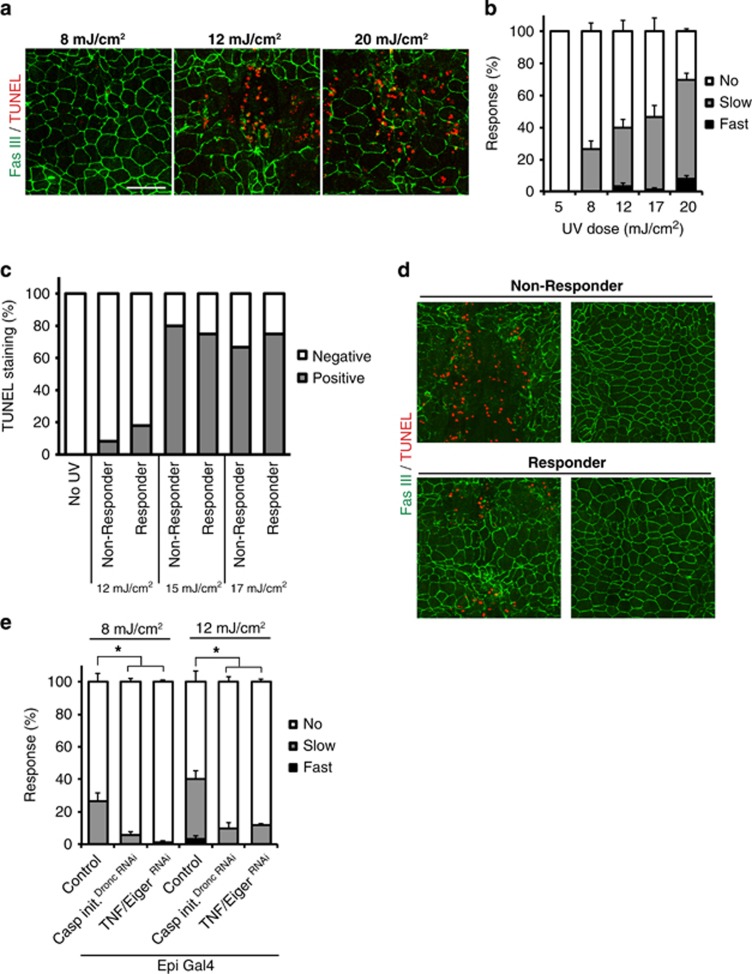
Procedural separation of cell death and thermal allodynia. (**a**) Epidermal morphology changes and TUNEL staining with different doses of UV, 24 h post irradiation. Anti-Fasciclin-3 (membranes, green) and TUNEL labeling (apoptotic cells, red). Bar, 50 *μ*m. (**b**) Dependence of UV-induced thermal allodynia upon various doses of UV, 24 h post irradiation. (**c**) Presence of TUNEL labeling in responsive or non-responsive larvae. *n*=9–12 in each group. (**d**) Epidermal morphology changes and TUNEL staining of responders and non-responders 24 h after 17 mJ/cm^2^ UV irradiation. Anti-Fasciclin-3 (membranes, green) and TUNEL labeling (apoptotic cells, red). (**e**) UV-induced thermal allodynia when *dronc* (initiator caspase) and *eiger* (TNF) were inhibited. *UAS-RNAi* lines are indicated

As an alternative test of whether cell death is necessary for thermal allodynia, we examined whether larvae exhibit nociceptive sensitization when treated with sub-apoptotic UV doses. To determine the lowest dose of UV that causes apoptosis, we examined epidermal TUNEL labeling in larvae exposed to decreasing UV.^13^ UV doses >12 mJ/cm^2^ caused both morphological disruption and positive TUNEL staining in epidermal cells, whereas doses <8 mJ/cm^2^ did not (Figure 2a). However, 8 mJ/cm^2^ – a UV dose that did not provoke overt apoptotic cell death – caused an attenuated but still substantial thermal allodynia response (Figure 2b). This response increased to ~70% at 20 mJ/cm^2^, the highest UV dose that ensures complete larval survival.^13^ To test whether cell death and thermal allodynia are correlated within individual larvae, we separated behavioral responders and non-responders and tested each group for epidermal TUNEL staining. We found TUNEL-positive larvae in both responder and non-responder groups (Figures 2c and d), suggesting that the presence or absence of cell death did not predict responder or non-responder status or vice versa. Regardless of whether apoptosis was present, Dronc and TNF/Eiger were still required for the UV-induced thermal allodynia (Figure 2e), suggesting that there is not a distinct signaling mechanism that induces thermal allodynia when apoptosis is absent. These data further suggest that the signal(s) that activate nociceptive sensitization can be produced even in the absence of overt cell death and that Dronc can promote these signal(s) independently of apoptotic activation.

### Canonical TNF/Eiger signaling factors are required within nociceptive sensory neurons for UV-induced thermal allodynia

**Figure 3:**
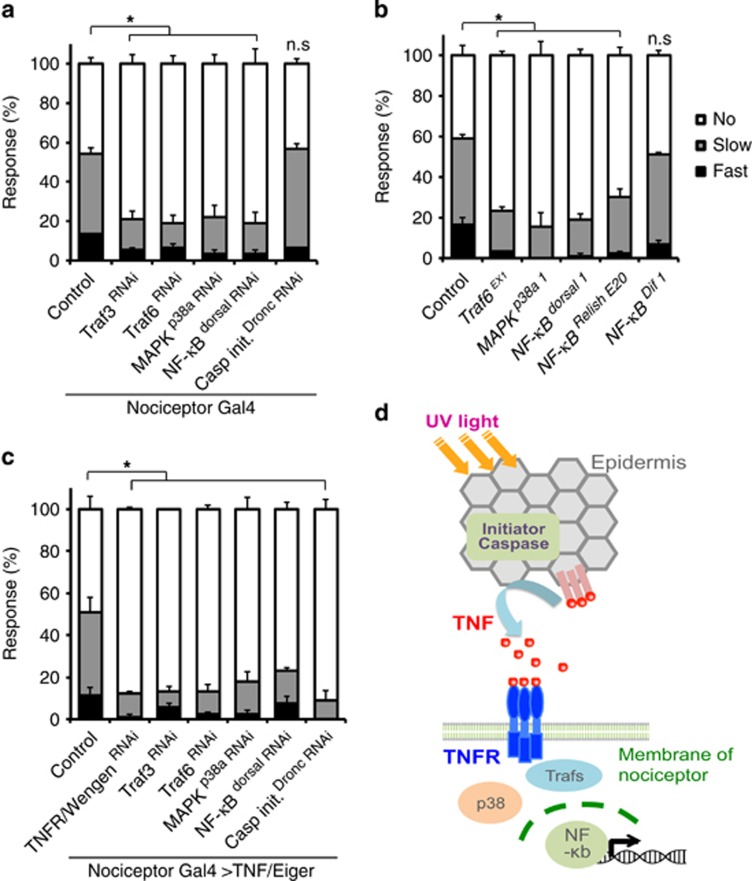
Downstream mediators of TNFR during thermal allodynia. (**a**) Thermal allodynia 24 h after UV irradiation. *UAS-RNAi* transgenes are indicated. (**b**) Thermal allodynia 24 h after UV irradiation in indicated mutants. (**c**) TNF/Eiger-induced genetic thermal allodynia on expression of the indicated RNAi transgenes. (**d**) Schematic model of TNF/TNFR signal transduction relevant to nociceptive sensitization

TNF/Eiger and its receptor TNFR/wengen are required for UV-induced allodynia.^13^ However, how the TNF/Eiger signal for thermal allodynia is transduced downstream of TNFR in sensory neurons is not known. To find downstream mediators of TNF/Eiger signaling during thermal allodynia, we focused on candidate downstream factors implicated in TNF/Eiger signaling pathway during immune and inflammatory responses.^43^ These included TRAFs, MAPKs, and NF-*κ*B transcription factors.^44^ We first tested nociceptive sensory neuron-specific expression (*ppk1.9-Gal4* (ref. 45)) of *UAS-RNAi* transgenes targeting *Traf3* and *Traf6*, *p38a* (a MAP kinase), and the NF-*κ*B-like transcription factor *dorsal* for UV-induced thermal allodynia. Expression of *UAS-RNAi* transgenes targeting each of these factors decreased thermal allodynia relative to the Gal4 driver and UAS-alone controls (Figure 3a; Supplementary Figure 3). Importantly, targeting of Dronc in sensory neurons (using the same *UAS-RNAi* transgene that is effective in the epidermis)^13^ did not interfere with thermal allodynia, demonstrating that Dronc functions specifically in the epidermis during development of UV-induced thermal allodynia (Figure 3a). To rule out RNAi off-target effects, we tested null mutants for *Traf6* (ref. 46) and *p38a* (ref. 47). These also show significant decreases in withdrawal behavior in comparison with control larvae (Figure 3b). As there are three NF-*κ*B homologs in *Drosophila* (*dorsal*, *Relish*, and *Dif*),^48, 49, 50^ we tested mutants of all of them. Null mutants for *dorsal* and *Relish* failed to develop thermal allodynia, but null mutants for *Dif* exhibited normal thermal allodynia after UV (Figure 3b). Nociceptive sensory neuron-specific expression of RNAi transgenes targeting these downstream mediators did not alter baseline thermal nociception (Supplementary Figure 3).

Ectopic overexpression of TNF/Eiger in nociceptive sensory neurons is sufficient to cause genetically- induced thermal allodynia in the absence of UV.^13^ Thus, we examined whether the expression of UAS-RNAi transgenes targeting *Traf3*, *Traf6*, *p38a*, and *dorsal* can attenuate the ectopic allodynia caused by TNF/Eiger overexpression. As expected, expression of *UAS-TNFR/Wengen*^*RNAi*^ attenuated TNF-induced thermal allodynia (Figure 3c). Likewise, expression of RNAi transgenes targeting *Traf3*, *Traf6*, *p38a*, and *dorsal* also reduced TNF-induced thermal allodynia, suggesting these factors are downstream mediators of TNF that are utilized to control nociceptive sensitization. Interestingly, Dronc, which is not required within nociceptive sensory neurons for UV-induced nociceptive sensitization (Figure 3a), is required for TNF-induced thermal allodynia (Figure 3c). This requirement of Dronc is specific to TNF-mediated sensitization as expression of *UAS-RNAi* transgenes targeting Dronc did not block genetic sensitization mediated by activation of either Hedgehog^51^ or Tachykinin^52^ signaling (Supplementary Figure 4). Our results suggest that Dronc may be required for some aspect of generating functional TNF/Eiger within the cell and that TNF/Eiger then acts through specific downstream mediators to affect nociceptive sensitization (Figure 3d).

### Dronc requires TNF/Eiger but not effector caspases to produce thermal allodynia

**Figure 4:**
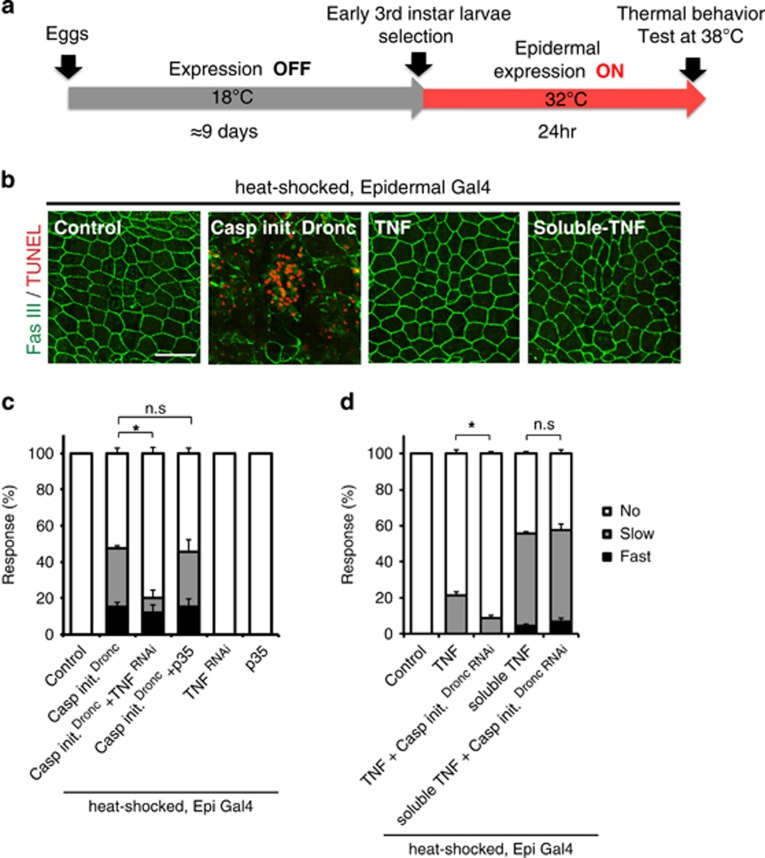
Dronc activates TNF signaling by regulating active TNF ligand production. (**a**) Schematic of TNF/Dronc activation experiments. *A58-Gal4*-mediated transgene expression was controlled by *tub-Gal80*^*ts*^. (**b**) Epidermal staining of indicated genotypes. Anti-Fasciclin-3 antibody (membranes, green) and TUNEL labeling (apoptotic cells, red) were used. Bar, 50 *μ*m. (**c**) Behavioral responses to 38 °C upon overexpression of Dronc with the expression of *UAS-TNF/Eiger*^*RNAi*^ and *UAS-p35*. (**d**) Thermal allodynia when full-length and soluble TNF/Eiger were overexpressed in the epidermis with or without the expression of an RNAi transgene targeting Dronc

As we found that epidermal Dronc is required for UV-induced thermal allodynia, we tested whether the overexpression of Dronc could cause ectopic thermal allodynia in the absence of UV irradiation. Because the activation of Dronc causes apoptotic cell death, we employed the *tub-Gal80*^*ts*^ system^53^ to avoid prior developmental defects (Figure 4a). Conditional overexpression of Dronc caused strong ectopic thermal allodynia as well as extensive apoptotic cell death in larval epidermis (Figures 4b and c). We hypothesized that if epidermal Dronc activates TNF/Eiger signaling to produce thermal allodynia, then expression of *UAS-RNAi* transgenes targeting TNF/Eiger would block the ectopic thermal allodynia induced by Dronc overexpression. Indeed, *UAS-TNF/Eiger*^*RNAi*^ expression significantly attenuated Dronc-induced ectopic thermal allodynia (Figure 4c). By contrast, overexpression of the effector caspase inhibitor p35 did not alter the ectopic thermal allodynia induced by Dronc overexpression (Figure 4c). Taken together, it appears that a Dronc-mediated non-apoptotic step in TNF/Eiger regulation is critical to induce thermal allodynia in *Drosophila* larvae.

### Soluble TNF/Eiger does not require Dronc to induce thermal allodynia

In mammalian cells, the ectodomain of TNF*α* can be secreted from the membrane following proteolytic cleavage by TNF*α*-converting enzyme.^54, 55^ Proteolytic processing of TNF/Eiger has also been reported in *Drosophila* S2 cells.^56^ Therefore, epidermal processing/secretion of TNF/Eiger could be required to activate sensory neuron TNFR following UV exposure. As overexpression of soluble TNF/Eiger in the epidermis was lethal, temporal overexpression of the relevant TNF/Eiger variants was induced using *tub-Gal80*^*ts*^ and heat shock (Figure 4a). When larvae were raised at the permissive temperature (18 °C), there was no thermal allodynia (Supplementary Figure 5). In the absence of UV irradiation, conditional epidermal overexpression of full-length TNF/Eiger did not cause strong ectopic allodynia (Figure 4d). By contrast, conditional epidermal overexpression of a processed soluble form of TNF/Eiger produced robust ectopic thermal allodynia (Figure 4d). There was no thermal allodynia in heat-shocked control larvae and no apoptotic cell death in the epidermis upon overexpression of soluble TNF/Eiger (Figures 4b and d). Consistent with the idea that Dronc might be required for some aspect of TNF processing or trafficking, expression of *UAS-Dronc*^*RNAi*^ did not block the ectopic allodynia induced by soluble TNF/Eiger overexpression (Figures 4d and 3a). This suggests that Dronc functions upstream of TNF/Eiger production.

### UV irradiation promotes TNF/Eiger production

The relative contribution(s) of potential transcriptional, translational, processing/secretion, or other regulatory mechanisms in regulating TNF activity during UV-induced thermal allodynia remain unclear. To understand how TNF/Eiger is regulated in the epidermis, we monitored changes in a transcriptional reporter^57^ following UV treatment. We found no detectable induction of this reporter following UV irradiation (Supplementary Figure 6).

**Figure 5:**
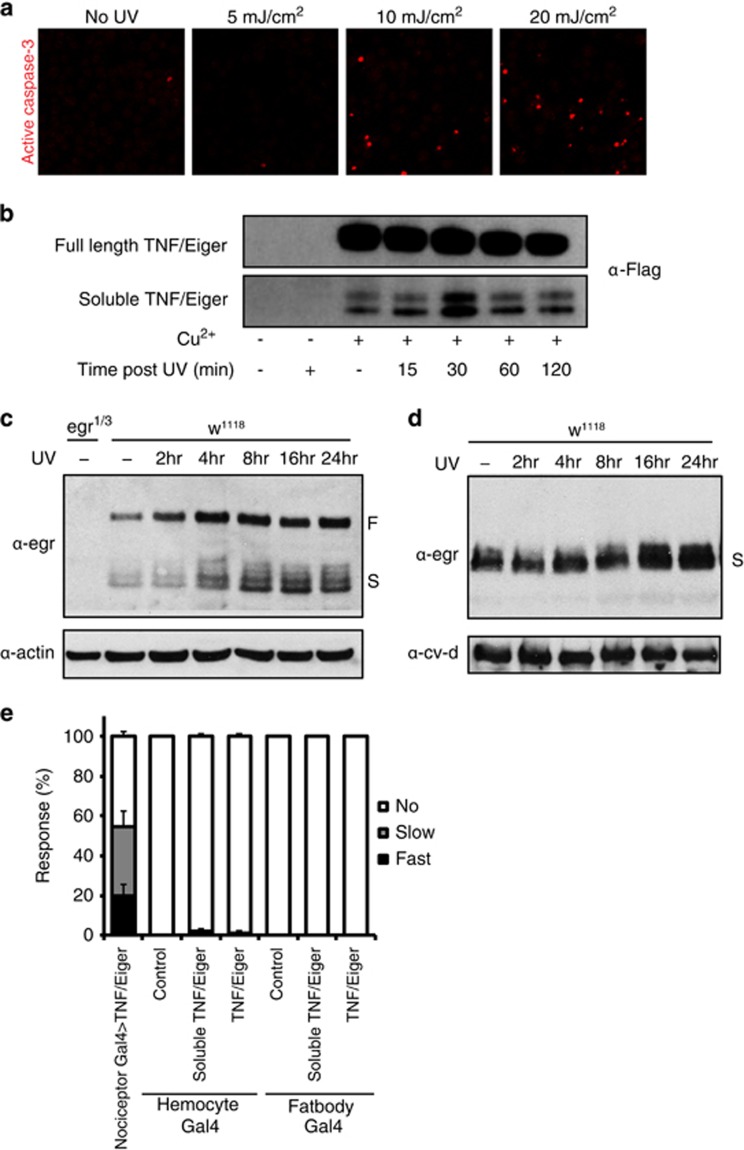
Secretion of soluble TNF/Eiger is constitutive and is enhanced by UV irradiation. (**a**) S2 cell apoptotic death in control and at different doses of UV. Anti-active caspase-3 staining (red). (**b**) Western blot analysis of S2 cells showing full-length TNF/Eiger from cell lysate and soluble TNF/Eiger from media supernatant. Induction by Cu^2+^ and UV treatment are indicated. (**c**) Western blot analysis of whole larval extracts with anti-TNF/Eiger at different times following UV irradiation. (**d**) Western blot analysis of hemolymph extracts with anti-TNF/Eiger. Time after UV irradiation is indicated. (**e**) Thermal allodynia (38 °C probe) upon ectopic expression of either full-length or soluble TNF/Eiger in hemocytes or fat body in the absence of UV. Genotypes are indicated

We next tested biochemically whether UV irradiation promotes increased processing or secretion of TNF/Eiger. We first monitored TNF/Eiger levels in *Drosophila* S2 cells. Active caspase-3 staining was performed 24 h after UV irradiation and 5 mJ/cm^2^ was selected as a sub-apoptotic dose (Figure 5a). As expected from previous studies on TNF/Eiger,^56, 58^ two different forms of TNF/Eiger proteins were detected in cell lysate and media respectively, indicating that processing of TNF/Eiger can occur spontaneously (Figure 5b). However, increased secretion of smaller-sized TNF/Eiger proteins into media was detected 30 min after UV irradiation in comparison with non-irradiated control (Figure 5b). These data suggest that UV treatment can trigger the production of soluble TNF/Eiger.

To see whether UV triggers production of TNF/Eiger *in vivo*, we monitored TNF levels in whole larval extracts following UV irradiation. Both full-length and soluble TNF/Eiger were induced (Figure 5c). Unlike S2 cells, which showed a rapid and transient increase of soluble TNF/Eiger (Figure 5b), *in vivo* TNF/Eiger levels were increased gradually; full-length TNF/Eiger started increasing 4 h after UV and remained up through 24 h, whereas soluble TNF/Eiger level peaked 8–16 h after UV. This increase was also observed in hemolymph extracts, with similar kinetics (Figure 5d). Previously, soluble TNF/Eiger was detected in hemolymph when its expression was driven in fat body.^58^ When we ectopically expressed either full-length or soluble TNF/Eiger from the fat body^59^ or hemocytes,^60^ we did not observe genetic allodynia in the absence of UV compared to the neuronal positive control (Figure 5e). This suggests that soluble TNF/Eiger in the hemolymph is not sufficient to induce thermal nociceptive sensitization.

### Identification of target genes upregulated during TNF-mediated nociceptive sensitization: validation of E(z)

**Figure 6:**
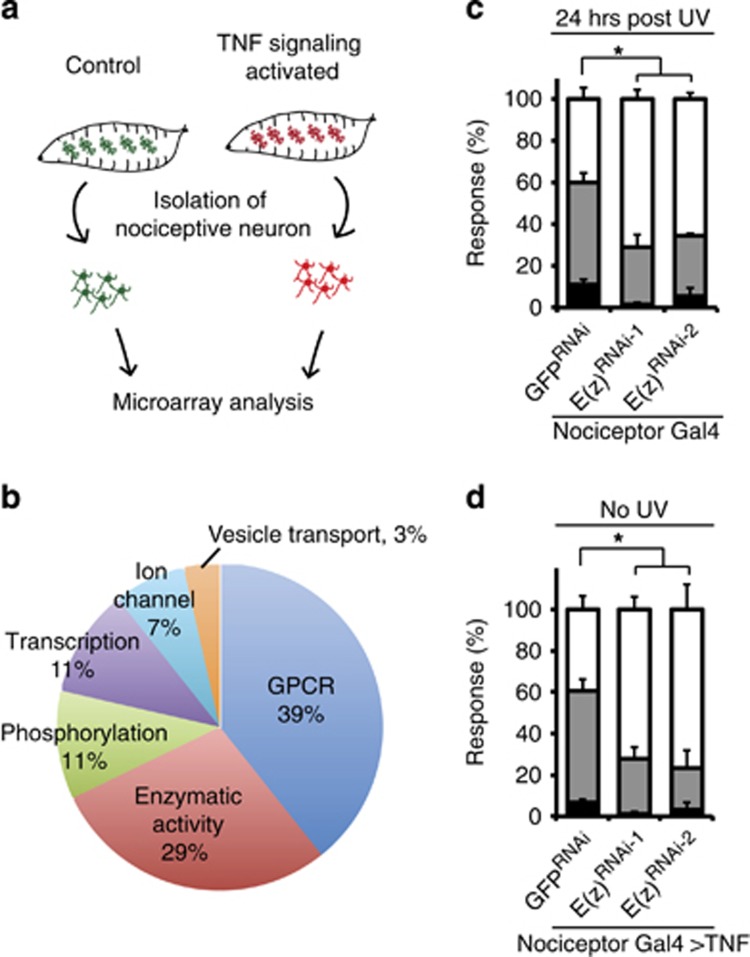
Microarray and behavioral analysis reveals E(z) as a functional downstream target gene of TNF/Eiger signaling. (**a**) Schematic of isolation of nociceptive sensory neurons and microarray analysis. (**b**) Categories of genes that have clear human orthologs, and that are upregulated in nociceptive sensory neurons that overexpress TNF/Eiger. (**c**) Quantification of UV-induced thermal allodynia on the expression of *UAS-E(z)*^*RNAi*^ transgenes in nociceptive sensory neurons. (**d**) Quantification of TNF/Eiger-induced thermal allodynia on the expression of *UAS-E(z)*^*RNAi*^ transgenes in nociceptive sensory neurons

To find target genes whose levels might be regulated by TNF signaling activation in sensory neurons, we performed cell-type-specific microarray analysis. Nociceptive sensory neurons were isolated from control larvae and from genetically- sensitized larvae by overexpressing TNF/Eiger in nociceptive sensory neurons (Figures 3c and 6a). We found 49 genes that were upregulated (>2.0-fold with *P*<0.01) in TNF signaling-activated sensory neurons compared to controls. Twenty-eight of these have clear human orthologs (Supplementary Table 1). These latter upregulated genes include G-protein-coupled receptors, enzymes including kinases, transcription factors, and ion channels (Figure 6b). Among these genes was a polycomb complex 2 gene, *E(z)*.^61^

*E(z)* encodes a histone lysine methyltransferase and is induced 2.6-fold compared to controls. We hypothesized that increased expression of *E(z)* is functionally important for nociceptive sensitization. Expression of two *UAS-E(z)*^*RNAi*^ transgenes in nociceptive sensory neurons was tested during both UV-induced and TNF/Eiger overexpression-induced thermal allodynia. Attenuated responses were observed in both types of thermal allodynia, suggesting that E(z) functions as an important target gene of TNF signaling in the development of thermal allodynia (Figures 6c and d). As the tested RNAi transgenes target non-overlapping portions of *E(z)*, off-target effects are highly unlikely (Figure 6). Nociceptive sensory neuronal expression of *UAS-E(z)*^*RNAi*^ caused a mild defect in baseline thermal nociception (Supplementary Figures 7a and b). Although it is required for dendritic arborization of a subtype of sensory neurons,^62^ expression of *UAS-E(z)*^*RNAi*^ transgenes did not alter the dendritic morphology of nociceptive sensory neurons (Supplementary Figure 7c). Taken together, our results identify and validate a novel target gene of TNF signaling during tissue damage-induced nociceptive sensitization.

## Discussion

We describe a novel apoptosis-independent role of Dronc in nociceptive sensitization. Five lines of experimental evidence support this: (1) UV-induced thermal allodynia is completely genetically separable from UV-induced apoptosis. (2) A low dose of UV that does not cause apoptosis still results in Dronc-dependent thermal allodynia. (3) UV irradiation can trigger cellular production of soluble TNF/Eiger. (4) Soluble TNF/Eiger-induced thermal alloydnia does not require Dronc function. (5) Ectopic expression of TNF/Eiger in sensory neurons reveals a new requirement for Dronc in the genetically-induced thermal allodynia.

How does Dronc function in producing active TNF/Eiger during sensitization? TNF/Eiger does not have a consensus caspase recognition site and any cleavage that would liberate extracellular TNF should necessarily occur outside of the cell. Thus, it is unlikely that Dronc cleaves TNF/Eiger directly. Alternatively, given the evidence that subcellular trafficking is important for TNF production,^63, 64^ Dronc could act on some component of the exocytic pathway to alter subcellular localization of TNF/Eiger. Alternatively, the irradiated epidermis might release Dronc itself and this extracellular Dronc could activate TNF/Eiger. There is precedence for extracellular caspase-6 release from axonal terminals that regulates TNF*α* secretion from microglial cells in the spinal cord.^65^

Inflammatory caspases mediate production of active cytokines such as interleukin-1*β*, forming inflammasome complexes that cleave the cytokine prodomain.^66, 67, 68^ Inflammasome complexes have been identified in mice, fish, and humans but not in invertebrates.^69^ How then do invertebrates orchestrate cytokine-dependent inflammatory responses? Our work might shed new light on this evolutionary problem. The *Drosophila* apoptosome may be functionally equivalent to an inflammasome.^68^ Indeed, Dronc is most similar to human caspase-2, which, although it functions as an apoptotic initiator, is also closely related to the inflammatory caspase subgroup.^39, 69^ Our data reveal that activation of the *Drosophila* apoptosome (Dark/Apaf-1 and Dronc) results in both apoptosis and production of active TNF/Eiger. This suggests that the *Drosophila* apoptosome may moonlight as an ancient version of the inflammasome.

Although the specific mechanism is as yet unclear, both full-length and soluble forms of TNF/Eiger were increased following UV treatment – transiently in S2 cells and in a more sustained manner in whole larvae. This suggests that UV increases both full-length expression and processing to a soluble form. Interestingly, local TNF/Eiger production in the epidermis appears more important for thermal allodynia, as overexpression of soluble TNF/Eiger in the epidermis was sufficient to induce ectopic allodynia, whereas hemolymph overexpression of soluble TNF/Eiger was not. Epidermal production of TNF/Eiger following UV irradiation may differ from what occurs during CFA-induced inflammatory pain in vertebrates. Here local TNF is thought to mainly derive from inflammatory cells.^29^ Interestingly, however, UV irradiation causes both sensitization in rats^9^ and production of TNF by epidermal keratinocytes.^70^

Our third main finding regards how TNFR/Wengen transduces TNF signaling to mediate thermal nociceptive sensitization. Thermal allodynia requires a number of canonical TNF signaling factors, including 2 TRAFs, a p38a MAP kinase and an NF-*κ*B-like transcription factor. In vertebrates, TRAF6 and p38 have been implicated in TNF*α*-induced mechanical hypersensitivity in astrocytes and DRG neurons, respectively.^71, 72^ In mouse sensory neurons, TRPV1 is required downstream of p38 for an electrophysiological correlate of sensitization,^72^ suggesting that phosphorylation of painless, the TRP channel required for thermal allodynia,^51^ is a plausible mechanism of sensitization *in vivo* in *Drosophila*. NF-*κ*B p50 subunit knockout mice are defective in both acute nociception and inflammatory sensitization,^73^ but the specific cell(s) in which this activity is required are not yet clear.

Because Dorsal functions downstream of TNF/Eiger signaling in sensory neurons during nociceptive sensitization, this NF-*κ*B factor may mediate transcriptional regulation of neuronal target genes. We have here developed a methodology for identifying and validating TNF-activated target genes in this context. *E(z)* is induced 2.6-fold by TNF expression in nociceptive sensory neurons and is required for nociceptive sensitization. TNF has been found to activate EZH2, the human ortholog of E(z), in skeletal myogenesis.^74^ Functional roles for polycomb group genes have not been reported in nociceptive biology although histone acetylation has been implicated in injury-induced alterations in nociceptive sensory neurons.^75^ Conditional knockout mouse lacking histone deacetylase (HDAC) 4 exhibit a specific decrease in thermal sensitization and TRPV1 expression.^76^ Increased global histone acetylation and epigenetic suppression through HDAC are also involved in inflammatory pain.^77^ The mechanism by which E(z) contributes to nociceptive sensitization is not yet clear but presumably involves its histone lysine methylation activity. Our data highlight the utility of the *Drosophila* model for identifying new conserved regulators of nociceptive sensitization and suggest that polycomb group gene members and histone methylation may be evolutionarily conserved regulators of nociceptive sensitization.

## Materials and methods

### UV treatment and behavioral analysis

UV treatment and thermal allodynia analysis were performed as previously described.^13^ Briefly, a UV crosslinker (Spectronics Corporation, Westbury, NY, USA) was pre-warmed for 100 s in a time-mode and right after warming up was over, larvae were irradiated in an energy-mode with 20 mJ/cm^2^ setting. A UV photometer (Spectronics Corporation) was used to measure actual amount of UV that the UV crosslinker emitted at each setting. Comparison between the UV crosslinker setting and the actual reading with a photometer is shown in Supplementary Table S2.

For nociceptive behavior analyses, a custom-designed heat probe was used as described previously.^13^ Washed larvae were placed under a Leica (Buffalo Grove, IL, USA) MZ6 light microscope and stimulated on their mid-dorsal side (third or fourth segment from the head) for a maximum of 20 s or until initiation of withdrawal behavior. Aversive withdrawal was defined as a 360° rolling along the anterior posterior body axis within 20 s of physical contact with the probe. Thermal allodynia was tested at 38 °C, 24 h after UV irradiation, whereas baseline nociception was tested at both 45 and 48 °C in the absence of injury. Behavioral responses were categorized as follows: fast (up to 5 s), slow (between 6 and 20 s), and no response (no response within 20 s). *χ*^2^-test was used to measure statistical significance in categorical data.

### Immunohistochemistry and apoptotic cell labeling

Dissection and immunostaining of larval epidermis were performed as previously described.^52^ Primary antibodies: anti-Fasciclin III (Developmental Studies Hybridoma Bank, Iowa City, IA, USA, 1:50), anti-activated Caspase-3 (Cell Signaling, Danvers, MA, USA, 1:150), and anti-GFP (Life Technologies, Basel, Switzerland, 1:500). Secondary antibodies: alexa488-conjugated anti-mouse IgG (Life Technologies, 1:1000), Cy3-conjugated goat anti-rabbit IgG (Jackson ImmunoResearch, West Grove, PA, USA, 1:1000). TUNEL labeling kit (Roche, Basel, Switzerland) was used to label apoptotic cells.

### Western blot analyses

S2 cells were transfected with Nextfect (Bio Scientific, Phoenix, AZ) or lipofectin (Invitrogen, Basel, Switzerland) transfection reagent to overexpress TNF/Eiger via pMT vector.^56^ Twnety-four hours after transfection, 50 μM of CuSO_4_ was added to induce expression. Twenty-four hours later, medium was removed, UV irradiation was performed, and fresh medium added. Cells and media were separated by centrifugation and cells were lysed in RIPA buffer (25 mM Tris/HCl (pH 7.6), 150 mM NaCl, 1% NP-40, 1% sodium deoxycholate, 0.1% SDS) containing protease inhibitor cocktail (Roche). Proteins in medium were concentrated using Vivaspin15R (Sartorius, Goettingen, Germany) in the presence of protease inhibitor. Lysate and concentrated medium were mixed with anti-Flag M2 affinity gel (Sigma-Aldrich, St. Louis, MO, USA) and incubated at 4 °C overnight. Flag gel beads were collected by centrifugation, washed with RIPA buffer, and boiled with 2 × sample buffer for 5 min. Supernatants were run on SDS-PAGE gels. Immunoblotting was performed using anti-Flag antibody (1:1000) conjugated with HRP (Cell signaling).

Whole larval extract was prepared by homogenizing PBS-washed larvae in RIPA buffer containing protease inhibitor cocktail. Protein concentration was measured (Bradford assay), and samples were boiled with 3 × sample buffer for 10 min and run on SDS-PAGE gels. Immunoblotting was performed using Anti-Egr (1:100),^58^ Anti-cv-d (1:2000),^78^ Anti-actin (1 : 5000, C4, MP Biomedicals, Santa Ana, CA, USA), HRP-conjugated anti-mouse IgG (Jackson ImmunoResearch), and HRP-conjugate anti-guinea pig IgG (Jackson ImmunoResearch) antibodies. Detection was with ECL reagents (Amersham, GE Healthcare Life Sciences, Little Chalfont, UK).
