# Supplementary material for: Drosophila caspase activity is required independently of apoptosis to produce active TNF/Eiger during nociceptive sensitization
Source: Cell Death Dis. 2017 May 11;8(5):e2786–. doi: 10.1038/cddis.2016.474 (PMC5520682; doi:10.1038/cddis.2016.474)
Supplement: Supplementary Information [file cddis2016474x1.pdf]

Supplementary Information

Fly Strains and Genetics

Fly stocks were maintained at 25°C and early third instar larvae were selected for UV treatment 4~5 days after copulation. *w*^{1118} was used as a control strain.[1] Null or hypomorphic alleles were used for behavioral tests: *egr*^{1} and *egr*^{3} served to inhibit TNF signaling.[2] *Dronc*^{I29}, D*ark*^{G8}, *Dark*^{S7}, *Dark*^{H16}, *Drice*^{17}, *Drice*$^{\Delta 1}$, *Dcp-1*^{prev}, *hid*^{1}, *skl*^{e1}, *skl*^{e3}, *Dredd*^{EP14}, *Strica*^{4}, *Damm*^{f02209}, *Decay*$^{\Delta K2}$, *Decay*$^{\Delta S3}$ were used to inhibit cell death signaling pathway.[3, 4, 5, 6, 7, 8, 9, 10, 11, 12] *Traf6*^{Ex1}, *p38a*^{1}, *dl*^{1}, *Rel*^{E20} and *Dif*^{1} were used to inhibit TNFR downstream.[13, 14, 15, 16, 17] *ppk-CD4-tdTom* was used to label nociceptive sensory neurons.[18] The Gal4/UAS system was employed to overexpress/knockdown transgenes in tissue specific manner. Gal4 drivers used: *A58 Gal4*[19] and *e22C Gal4*[20], *ppk1.9 Gal4*[21], *hmlΔ Gal4*[22], and *Gal4*^{OK376} (ref. 23). UAS-transgenes used: *UAS-regg*^{1} (full-length TNF)[2], *UAS-eiger60* (soluble TNF)[24], *UAS-Dronc*[25], *UAS-hid*[26], *UAS-grim*[27], and *UAS-reaper*[28] , *UAS-DIAP1*[26], *UAS-p35*[29], *UAS-ptcDN*[30], *UAS-DTKR-GFP*[31], UAS-eGFP (Bloomington), *UAS-miRHG.*[32] *UAS-DroncRNAi (8091R1) (*NIG), *UAS-EigerRNAi*[2], *UAS-WengenRNAi.*[33] *UAS-RNAi* lines from Vienna *Drosophila* Research Center (VDRC)[34]: 21830 (grim), 12045 (reaper), 8269 (hid), 34836 (traf3), 16125 (traf6), 52277 (p38), 45998 (dorsal), 107072 (E(z)). *JF02826* (E(z)) and *UAS-GFPRNAi* (9930) were from Bloomington stock center. The precise genotypes for each figure panel in this study are listed in Supplemental information.

Microarray methodology

The isolation and purification of nociceptive sensory neurons from third instar larvae was performed as previously described.[35] Briefly, control larvae (*ppk1.9-GAL4,UAS-mCD8::GFP*) and larvae which overexpress TNF/Eiger (*ppk1.9-GAL4,UAS-mCD8::GFP; UAS-regg^{1}*) were used to isolate wild-type and genetically sensitized nociceptive sensory neurons via magnetic bead cell sorting. Isolated nociceptive neurons were then prepared for microarray gene expression analyses as previously described.[36] mRNA isolation, amplification, labeling, hybridization, and microarray analyses were performed by Miltenyi Biotec. 250 ng of each of the sample cDNAs were used as template for Cy3 (control) and Cy5 (TNF/Eiger overexpression) labeling. Labeled cDNAs were combined and hybridized to an Agilent whole *Drosophila* genome oligo microarray (4x44K V2) and analyses were conducted in quadruplicate. Microarray expression and bioinformatics analyses were performed as previously described.[36, 37] Normalized Cy5/Cy3 fold changes (Cy5/Cy3-log10 ratios) were used to investigate differentially expressed genes with a threshold fold change >2 (mean fold change of genes across four replicate arrays) and a p-value <0.01 for genes that are up-regulated in TNF signaling-activated sensory nociceptive neurons relative to controls.

Supplementary Figure legends

Supplementary Figure 1 Pro-apoptotic genes and other caspases are not required for both epidermal apoptosis and thermal allodynia by UV irradiation**.** (**a** and **b**) Larval epidermal staining of indicated genotypes. (**a**) Grim and Reaper overexpression in the larval epidermis does not induce apoptosis. (**b**) Localized and temporally-controlled expression of UAS-Hid via Pannier-Gal4 can induce apoptosis. Anti-Fasciclin-3 antibody

(membranes, green) and anti-activated Caspase3 labeling (apoptotic cells, red) were used. (**c**) Larval epidermal staining 24 h after UV irradiation. Anti-Fasciclin-3 antibody (membranes, green) and TUNEL labeling (apoptotic cells, red) were used. (**d** and **e**) Measurement of UV-induced thermal allodynia at 38 °C, 24 h post UV irradiation. Genotypes are indicated. n = 3 sets of 30 larvae. Error bars represent SEM.

Supplementary Figure 2 Baseline thermal nociception analysis for genotypes relevant to Figure 1 and UAS-only controls for Figure 1D. (**a-d**) Measurement of aversive withdrawal behavior of indicated genotypes at 45°C (**a** and **b**) or 48°C (**c** and **d**) in the absence of tissue damage. (**e**) UV-induced thermal allodynia of indicated *UAS-transgene* alone control at 38 °C.

n = 3 sets of 30 larvae. Error bars represent SEM.

Supplementary Figure 3 Baseline thermal nociception analysis for genotypes relevant to Figure 3. (**a-d**) Measurement of aversive withdrawal behavior of indicated genotypes at 45°C (**a** and **c**) or 48°C (**b** and **d**) in the absence of tissue damage.

n = 3 sets of 30 larvae. Error bars represent SEM.

Supplementary Figure 4 Expression of *UAS-Dronc^{RNAi}* does not inhibit thermal allodynia induced by hyperactivation of Hedgehog or Tachykinin signaling. UV-induced thermal allodynia of indicated genotypes alone control at 38 °C. n = 3 sets of 30 larvae. Error bars represent SEM.

Supplementary Figure 5 Non-heat-shocked larvae of the genotypes relevant to Figure 4 do not show thermal allodynia. Measurement of aversive withdrawal behavior at 38 °C when larvae of the indicated genotypes were maintained at the non-permissive (no heat shock) temperature (18°C). n = 3 sets of 30 larvae. Error bars represent SEM.

Supplementary Figure 6 A TNF/Eiger transcriptional reporter is not induced by UV irradiation. Epidermal whole mounts in larvae bearing *UAS-GFP* under control of *TNF/Eiger-Gal4* after UV irradiation (Anti-GFP, green).

Supplementary Figure 7 Baseline thermal nociception of larvae expressing *UAS-E(z)*^{RNAi} in nociceptive sensory neurons. (**a** and **b**) Measurement of aversive withdrawal behavior of larvae of the indicated genotypes at 45 °C (**a**) or 48 °C (**b**). n = 3 sets of 30 larvae. Error bars represent SEM. (**c**) Dendritic morphology of nociceptive sensory neurons in larvae indicated genotype.

Genotypes of flies used in this study

Figure 1b

w^{1118};
w^{1118};
w^{1118};;$dronc^{I29}$
w^{1118};$dark^{G8}$/$dark^{H16}$
w^{1118};;$drice^{\Delta 1}$
w^{1118};$dcp\text{-}1^{prev}$
w^{1118};*e22C Gal4/+*
w^{1118};*e22C Gal4/UAS-DIAP1.H*
w^{1118};*e22C Gal4/UAS-p35*

Figure 1c

w^{1118};
w^{1118};;$dronc^{I29}$

w^{1118};$dark^{G8}$/$dark^{H16}$
w^{1118};$dark^{G8}$/$dark^{S7}$
w^{1118};$dark^{S7}$/$dark^{H16}$
w^{1118};;$drice^{\Delta 1}$
w^{1118};$dcp\text{-}1^{prev}$
w^{1118};$dcp\text{-}1^{prev}$;$drice^{\Delta 1}$
w^{1118};$eiger^{1}$/+
w^{1118};$eiger^{3}$/+
w^{1118};$eiger^{1}$/$eiger^{3}$

Figure 1d, S2a, and S2c
w^{1118};;A58 Gal4/+
w^{1118};UAS-DIAP1.H/+; A58 Gal4/+
w^{1118};UAS-$Dronc^{RNAi}$ (8081R1)/+; A58 Gal4/+
w^{1118};e22C Gal4/+
w^{1118};e22C Gal4/UAS-p35
w^{1118};e22C Gal4/ UAS-$Dronc^{RNAi}$ (8081R1)

Figure 2a and 2b
w^{1118};

Figure 2c
w^{1118};;A58 Gal4/+
w^{1118};UAS-$Dronc^{RNAi}$ (8081R1)/+; A58 Gal4/+
w^{1118};UAS-$Eiger^{RNAi}$ /+; A58 Gal4/+
w^{1118};;A58 Gal4/+
w^{1118}; UAS-$Dronc^{RNAi}$ (8081R1)/+; A58 Gal4/+
w^{1118}; UAS-$Eiger^{RNAi}$ /+; A58 Gal4/+

Figure 3a, S3a, and S3b
w^{1118};;ppk1.9 Gal4/+
w^{1118};;ppk1.9 Gal4/ UAS-$Traf3^{RNAi}$(v34836)
w^{1118};UAS-$Traf6^{RNAi}$(v16125)/+;ppk1.9 Gal4/+
w^{1118};;ppk1.9 Gal4/UAS-$p38a^{RNAi}$(v52277)
w^{1118};;ppk1.9 Gal4/UAS-dl^{RNAi}(v45998)
w^{1118};UAS-$Dronc^{RNAi}$(8081R1)/+;ppk1.9 Gal4/+

Figure 3b
w^{1118};
w^{1118}, $traf6^{EX1}$;
w^{1118};;$p38a^{1}$
w^{1118};dl^{1}
w^{1118};;$relish^{E20}$
w^{1118};Dif^{1}

Figure 3c

w^{1118};regg^{1}/+;ppk1.9 Gal4/+
w^{1118}; regg^{1}/UAS-Wengen^{RNAi}/+;ppk1.9 Gal4/+
w^{1118}; regg^{1}/+;;ppk1.9 Gal4/ UAS-Traf3^{RNAi}(v34836)
w^{1118}; regg^{1}/UAS-Traf6^{RNAi}(v16125)/+;ppk1.9 Gal4/+
w^{1118}; regg^{1}/+;;ppk1.9 Gal4/UAS-p38a^{RNAi}(v52277)
w^{1118}; regg^{1}/+;;ppk1.9 Gal4/UAS-Dorsal^{RNAi}(v45998)
w^{1118}; regg^{1}/UAS-Dronc^{RNAi}(8081R1)/+;ppk1.9 Gal4/+

Figure 4b
w^{1118};tubGal80^{ts}/+;A58 Gal4/+
w^{1118};tubGal80^{ts}/+;A58 Gal4/UAS-Dronc
w^{1118};tubGal80^{ts}/regg^{1};A58 Gal4/+
w^{1118};tubGal80^{ts}/UAS-Eiger 60;A58 Gal4/+

Figure 4c and S5a
w^{1118};tubGal80^{ts}/+;A58 Gal4/+
w^{1118};tubGal80^{ts}/+;A58 Gal4/UAS-Dronc
w^{1118};tubGal80^{ts}/UAS-Eiger^{RNAi};A58 Gal4/UAS-Dronc
w^{1118};tubGal80^{ts}/UAS-p35;A58 Gal4/UAS-Dronc
w^{1118};tubGal80^{ts}/UAS-Eiger^{RNAi};A58 Gal4/+
w^{1118};tubGal80^{ts}/UAS-p35;A58 Gal4/+

Figure 4d and S5b
w^{1118};tubGal80^{ts}/+;A58 Gal4/+
w^{1118};tubGal80^{ts}/regg^{1};A58 Gal4/+
w^{1118};tubGal80^{ts}/regg^{1}, UAS-Dronc^{RNAi}(8081R1);A58 Gal4/+
w^{1118};tubGal80^{ts}/UAS-Eiger60;A58 Gal4/+
w^{1118};tubGal80^{ts}/UAS-Eiger60, UAS-Dronc^{RNAi}(8081R1);A58 Gal4/+

Figure 5c and 5d
w^{1118};

Figure 5e
w^{1118};regg^{1}/+;ppk1.9 Gal4/+
w^{1118};hmlΔ Gal4/+
w^{1118};hmlΔ Gal4/UAS-Eiger60
w^{1118};hmlΔ Gal4/regg^{1}
w^{1118};Gal4^{OK376}/+
w^{1118};Gal4 ^{OK376}/UAS-Eiger60
w^{1118};Gal4 ^{OK376}/regg^{1}

Figure 6c, S7a, and S7b
w^{1118};UAS-GFP^{RNAi}(9331);ppk1.9 Gal4/+
w^{1118};UAS-E(z)^{RNAi}(v107072);ppk1.9 Gal4/+
w^{1118};UAS-E(z)^{RNAi}(BL27993);ppk1.9 Gal4/+

Figure 6d
w^{1118};UAS-GFP^{RNAi}(9331)/regg^{1};ppk1.9 Gal4/+
w^{1118};UAS-E(z)^{RNAi}(v107072)/regg^{1};ppk1.9 Gal4/+
w^{1118};UAS-E(z)^{RNAi}(BL27993)/regg^{1};ppk1.9 Gal4/+

Figure S1a
w^{1118};;A58 Gal4/UAS-Grim
w^{1118};UAS-Reaper/+;A58 Gal4/+

Figure S1b
w^{1118};tubGal80^{ts}/+;pnr-Gal4,UAS-eGFP/+
w^{1118},UAS-Hid/+;tubGal80^{ts}/+;pnr-Gal4,UAS-eGFP/+

Figure S1c
w^{1118};dark^{G8}/dark^{S7}
w^{1118};dark^{S7}/dark^{H16}
;;hid^{1}
w^{1118};;sickle^{1}/ sickle^{3}
w^{1118};strica^{4}
w^{1118}, dredd^{EP1412};
w^{1118};;decay$^{\Delta K2}$
w^{1118};damm^{f02209}

Figure S1d
w^{1118};;A58 Gal4/+
w^{1118};UAS-RHG^{miRNA};A58 Gal4/+
w^{1118};UAS-Grim^{RNAi}(v21830)/+;A58 Gal4/UAS-Reaper^{RNAi}(v12045),UAS-Hid^{RNAi}(v8269)
w^{1118};UAS-DIAP1.H/+; A58 Gal4/+
w^{1118};UAS-Dronc^{RNAi} (8081R1)/+; A58 Gal4/+

Figure S1e
w^{1118};
w^{1118};dark^{G8}/dark^{S7}
w^{1118};dark^{S7}/dark^{H16}
;;hid^{1}
w^{1118};;sickle^{1}/ sickle^{3}
w^{1118};strica^{4}
w^{1118}, dredd^{EP1412};
w^{1118};;decay$^{\Delta K3}$
w^{1118};;decay$^{\Delta K2}$
w^{1118};damm^{f02209}

Figure S2b and S2d
w^{1118};
w^{1118};;dronc^{I29}

w^{1118}*;dark*^{G8}*/dark*^{H16}
w^{1118}*;dark*^{G8}*/dark*^{S7}
w^{1118}*;dark*^{S7}*/dark*^{H16}
w^{1118}*;;drice*^{17}

Figure S2e
w^{1118}*;UAS-p35/+*
w^{1118}*;UAS-DIAP1.H/+*
w^{1118}*;UAS-Dronc*^{RNAi} *(8081R1)/+*

Figure S3c and S3d
w^{1118}*;*
w^{1118}*, traf6*^{EX1}*;*
w^{1118}*;;p38a*^{1}
w^{1118}*;dorsal*^{1}
w^{1118}*;;relish*^{E20}

Figure S3e
w^{1118}*;;UAS-Traf3*^{RNAi}*(v34836)/+*
w^{1118}*;UAS-Traf6*^{RNAi}*(v16125)/+*
w^{1118}*;;UAS-p38a*^{RNAi}*(v52277)/+*
w^{1118}*;;UAS-Dorsal*^{RNAi}*(v45998)/+*

Figure S4
w^{1118}*;UAS-Ptc*^{DN}*/+;ppk1.9 Gal4/+*
w^{1118}*;UAS-Ptc*^{DN}*/UAS-Dronc*^{RNAi} *(8081R1);ppk1.9 Gal4/+*
w^{1118}*;UAS-DTKR-GFP/+;ppk1.9 Gal4/+*
w^{1118}*;UAS- DTKR-GFP/UAS-Dronc*^{RNAi} *(8081R1);ppk1.9 Gal4/+*

Figure S6
w^{1118}*;Eiger-Gal4/UAS-GFP*

Figure S7c
w^{1118}*;ppk-CD4TdTom/+;ppk1.9 Gal4/+*
w^{1118}*;ppk-CD4TdTom/regg*^{1}*;ppk1.9 Gal4/+*
w^{1118}*;ppk-CD4TdTom/ UAS-E(z)*^{RNAi}*(BL27993);ppk1.9 Gal4/+*

Supporting References

1. Rabinow L, Birchler JA. A dosage-sensitive modifier of retrotransposon-induced alleles of the Drosophila white locus. *The EMBO journal* 1989, **8**(3): 879-889.

2. Igaki T, Kanda H, Yamamoto-Goto Y, Kanuka H, Kuranaga E, Aigaki T*, et al.* Eiger, a TNF superfamily ligand that triggers the Drosophila JNK pathway. *The EMBO journal* 2002, **21**(12)**:** 3009-3018.

3. Xu D, Li Y, Arcaro M, Lackey M, Bergmann A. The CARD-carrying caspase Dronc is essential for most, but not all, developmental cell death in Drosophila. *Development* 2005, **132**(9)**:** 2125-2134.

4. Srivastava M, Scherr H, Lackey M, Xu D, Chen Z, Lu J*, et al.* ARK, the Apaf-1 related killer in Drosophila, requires diverse domains for its apoptotic activity. *Cell death and differentiation* 2007, **14**(1)**:** 92-102.

5. Muro I, Berry DL, Huh JR, Chen CH, Huang H, Yoo SJ*, et al.* The Drosophila caspase Ice is important for many apoptotic cell deaths and for spermatid individualization, a nonapoptotic process. *Development* 2006, **133**(17)**:** 3305-3315.

6. Laundrie B, Peterson JS, Baum JS, Chang JC, Fileppo D, Thompson SR*, et al.* Germline cell death is inhibited by P-element insertions disrupting the dcp-1/pita nested gene pair in Drosophila. *Genetics* 2003, **165**(4)**:** 1881-1888.

7. Lindsley DL, Zimm GG, Lindsley DL. *The genome of Drosophila melanogaster*. Academic Press: San Diego, 1992.

8. Lee G, Sehgal R, Wang Z, Nair S, Kikuno K, Chen CH*, et al.* Essential role of grim-led programmed cell death for the establishment of corazonin-producing peptidergic nervous system during embryogenesis and metamorphosis in Drosophila melanogaster. *Biology open* 2013, **2**(3)**:** 283-294.

9. Elrod-Erickson M, Mishra S, Schneider D. Interactions between the cellular and humoral immune responses in Drosophila. *Curr Biol* 2000, **10**(13)**:** 781-784.

10. Baum JS, Arama E, Steller H, McCall K. The Drosophila caspases Strica and Dronc function redundantly in programmed cell death during oogenesis. *Cell death and differentiation* 2007, **14**(8)**:** 1508-1517.

11. Kondo S, Senoo-Matsuda N, Hiromi Y, Miura M. DRONC coordinates cell death and compensatory proliferation. *Molecular and cellular biology* 2006, **26**(19)**:** 7258-7268.

12. Lee G, Wang Z, Sehgal R, Chen CH, Kikuno K, Hay B*, et al.* Drosophila caspases involved in developmentally regulated programmed cell death of peptidergic neurons during early metamorphosis. *J Comp Neurol* 2011, **519**(1)**:** 34-48.

13. Cha GH, Cho KS, Lee JH, Kim M, Kim E, Park J*, et al.* Discrete functions of TRAF1 and TRAF2 in Drosophila melanogaster mediated by c-Jun N-terminal kinase and NF-kappaB-dependent signaling pathways. *Molecular and cellular biology* 2003, **23**(22)**:** 7982-7991.

14. Craig CR, Fink JL, Yagi Y, Ip YT, Cagan RL. A Drosophila p38 orthologue is required for environmental stress responses. *EMBO reports* 2004, **5**(11)**:** 1058-1063.

15. Leiss D, Hinz U, Gasch A, Mertz R, Renkawitz-Pohl R. Beta 3 tubulin expression characterizes the differentiating mesodermal germ layer during Drosophila embryogenesis. *Development* 1988, **104**(4)**:** 525-531.

16. Hedengren M, Asling B, Dushay MS, Ando I, Ekengren S, Wihlborg M*, et al.* Relish, a central factor in the control of humoral but not cellular immunity in Drosophila. *Molecular cell* 1999, **4**(5)**:** 827-837.

17. Rutschmann S, Jung AC, Hetru C, Reichhart JM, Hoffmann JA, Ferrandon D. The Rel protein DIF mediates the antifungal but not the antibacterial host defense in Drosophila. *Immunity* 2000, **12**(5)**:** 569-580.

18. Han C, Jan LY, Jan YN. Enhancer-driven membrane markers for analysis of nonautonomous mechanisms reveal neuron-glia interactions in Drosophila. *Proceedings of the National Academy of Sciences of the United States of America* 2011, **108**(23)**:** 9673-9678.

19. Galko MJ, Krasnow MA. Cellular and genetic analysis of wound healing in Drosophila larvae. *PLoS biology* 2004, **2**(8)**:** E239.

20. Lawrence PA, Bodmer R, Vincent JP. Segmental patterning of heart precursors in Drosophila. *Development* 1995, **121**(12)**:** 4303-4308.

21. Ainsley JA, Pettus JM, Bosenko D, Gerstein CE, Zinkevich N, Anderson MG*, et al.* Enhanced locomotion caused by loss of the Drosophila DEG/ENaC protein Pickpocket1. *Curr Biol* 2003, **13**(17)**:** 1557-1563.

22. Sinenko SA, Mathey-Prevot B. Increased expression of Drosophila tetraspanin, Tsp68C, suppresses the abnormal proliferation of ytr-deficient and Ras/Raf-activated hemocytes. *Oncogene* 2004, **23**(56)**:** 9120-9128.

23. Lazareva AA, Roman G, Mattox W, Hardin PE, Dauwalder B. A role for the adult fat body in Drosophila male courtship behavior. *PLoS genetics* 2007, **3**(1)**:** e16.

24. Narasimamurthy R, Geuking P, Ingold K, Willen L, Schneider P, Basler K. Structure-function analysis of Eiger, the Drosophila TNF homolog. *Cell research* 2009, **19**(3)**:** 392-394.

25. Meier P, Silke J, Leevers SJ, Evan GI. The Drosophila caspase DRONC is regulated by DIAP1. *The EMBO journal* 2000, **19**(4)**:** 598-611.

26. Lohmann I, McGinnis N, Bodmer M, McGinnis W. The Drosophila Hox gene deformed sculpts head morphology via direct regulation of the apoptosis activator reaper. *Cell* 2002, **110**(4)**:** 457-466.

27. Chen P, Rodriguez A, Erskine R, Thach T, Abrams JM. Dredd, a novel effector of the apoptosis activators reaper, grim, and hid in Drosophila. *Developmental biology* 1998, **201**(2)**:** 202-216.

28. Aplin AC, Kaufman TC. Homeotic transformation of legs to mouthparts by proboscipedia expression in Drosophila imaginal discs. *Mechanisms of development* 1997, **62**(1)**:** 51-60.

29. Zhou L, Schnitzler A, Agapite J, Schwartz LM, Steller H, Nambu JR. Cooperative functions of the reaper and head involution defective genes in the programmed cell death of Drosophila central nervous system midline cells. *Proceedings of the National Academy of Sciences of the United States of America* 1997, **94**(10)**:** 5131-5136.

30. Johnson RL, Milenkovic L, Scott MP. In vivo functions of the patched protein: requirement of the C terminus for target gene inactivation but not Hedgehog sequestration. *Molecular cell* 2000, **6**(2)**:** 467-478.

31. Ignell R, Root CM, Birse RT, Wang JW, Nassel DR, Winther AM. Presynaptic peptidergic modulation of olfactory receptor neurons in Drosophila. *Proceedings of the National Academy of Sciences of the United States of America* 2009, **106**(31)**:** 13070-13075.

32. Siegrist SE, Haque NS, Chen CH, Hay BA, Hariharan IK. Inactivation of both Foxo and reaper promotes long-term adult neurogenesis in Drosophila. *Curr Biol* 2010, **20**(7)**:** 643-648.

33. Kanda H, Igaki T, Kanuka H, Yagi T, Miura M. Wengen, a member of the Drosophila tumor necrosis factor receptor superfamily, is required for Eiger signaling. *The Journal of biological chemistry* 2002, **277**(32)**:** 28372-28375.

34. Dietzl G, Chen D, Schnorrer F, Su KC, Barinova Y, Fellner M*, et al.* A genome-wide transgenic RNAi library for conditional gene inactivation in Drosophila. *Nature* 2007, **448**(7150)**:** 151-156.

35. Iyer EP, Iyer SC, Sulkowski MJ, Cox DN. Isolation and purification of Drosophil peripheral neurons by magnetic bead sorting. *Journal of visualized experiments : JoVE* 2009(34).

36. Bhattacharya S, Iyer EP, Iyer SC, Cox DN. Cell-type specific transcriptomic profiling to dissect mechanisms of differential dendritogenesis. *Genomics data* 2014, **2:** 378-381.

37. Iyer EP, Iyer SC, Sullivan L, Wang D, Meduri R, Graybeal LL*, et al.* Functional genomic analyses of two morphologically distinct classes of Drosophila sensory neurons: post-mitotic roles of transcription factors in dendritic patterning. *PLoS One* 2013, **8**(8)**:** e72434.

Supporting Table 1. Genes upregulated upon sensory neuronal TNF/Eiger overexpression.

CG number	Gene	Molecular Function	Human ortholog	Fold change	Sequence ID	Sequence code
CG31201	Glutamate receptor IIE	Ion channel	GRIK2	2.1630475	2499355	A_09_P059281
CG3772	cryptochrome	GPCR signaling	CRY1, CRY2	4.27233	2495248	A_09_P091155
CG1147	Neuropeptide F receptor	GPCR signaling	NPY5R	4.200895	2500094	A_09_P036656
CG33183	Hormone receptor-like in 46	Transcription	RORB	4.195125	2507060	A_09_P144940
CG18741	Dopamine receptor 2	GPCR signaling	ADRA1B, ADRA1D, HTR7	4.4514525	2504200	A_09_P030561
CG33344	Cardioacceleratory peptide receptor	GPCR signaling	NPSR1	2.2945625	2484477	A_09_P047356
CG34381	Trissin receptor	GPCR signaling	GPR139, GPR19, NPBWR1	3.1337875	2495952	A_09_P216970
CG31760		GPCR signaling	GPR179	2.1514775	2487965	A_09_P013641
CG7431	Tyramine receptor	GPCR signaling	ADRA1D, HRH1, HRH2, HTR4	3.85525	2488554	A_09_P214165
CG10888	Rhodopsin 3	GPCR signaling	OPN4	2.826415	2507755	A_09_P044391
CG33513	NMDA receptor 2	Ion channel	GRIN2D	2.1088125	2507487	A_09_P047611
CG7383	eagle	Transcription	VDR	1.895905	2492609	A_09_P135120
CG9918	Pyrokinin 1 receptor	GPCR signaling	NMUR1, NMUR2	2.5064225	2512044	A_09_P074941
CG15744		GPCR signaling	ADGRA2	2.6660225	2496091	A_09_P038331
CG6899	Protein tyrosine phosphatase 4E	Phosphatase, receptor	PTPRB	1.87305	2498522	A_09_P170990
CG2872	Allatostatin Receptor	GPCR signaling	GALR2	2.910375	2514720	A_09_P066211
CG4007	Neurospecific receptor kinase	Enymatic activity	MUSK	2.0737025	2516306	A_09_P032321
CG11783	Hormone receptor-like in 96	Transcription	NR1I2	3.283885	2488919	A_09_P030601
CG34384		Enymatic activity	DGKH	4.6278075	2496126	A_09_P147935
CG11111	retinal degeneration B	Enymatic activity	PITPNM2	3.2450125	2508957	A_09_P051071
CG2171	Triose phosphate isomerase	Enymatic activity	TPI1	2.66855	2489405	A_09_P146560
CG6502	Enhancer of zeste	Enymatic activity	EZH2	2.6339075	2491045	A_09_P042316
CG10986	garnet	Protein transport	AP3D1	3.28213	2493939	A_09_P104225
CG4747		Enymatic activity	GLYR1	4.1603975	2511026	A_09_P204720
CG2155	vermilion	Enymatic activity	TDO2	2.4186625	2514857	A_09_P009976
CG12529	Zwischenferment	Enymatic activity	G6PD	1.9283325	2511576	A_09_P010116
CG6728	ninaG	Enymatic activity	CHDH	3.8052075	2496597	A_09_P073811

Supporting Table 2. Comparison between instrument settings and measured- UV doses.

Set in UV crosslinker	Measured in UV radiometer
5 mJ/cm^2	2-3 mJ/cm^2
8 mJ/cm^2	4-5 mJ/cm^2
12 mJ/cm^2	6-7 mJ/cm^2
17 mJ/cm^2	8-10 mJ/cm^2
20 mJ/cm^2	11-14 mJ/cm^2

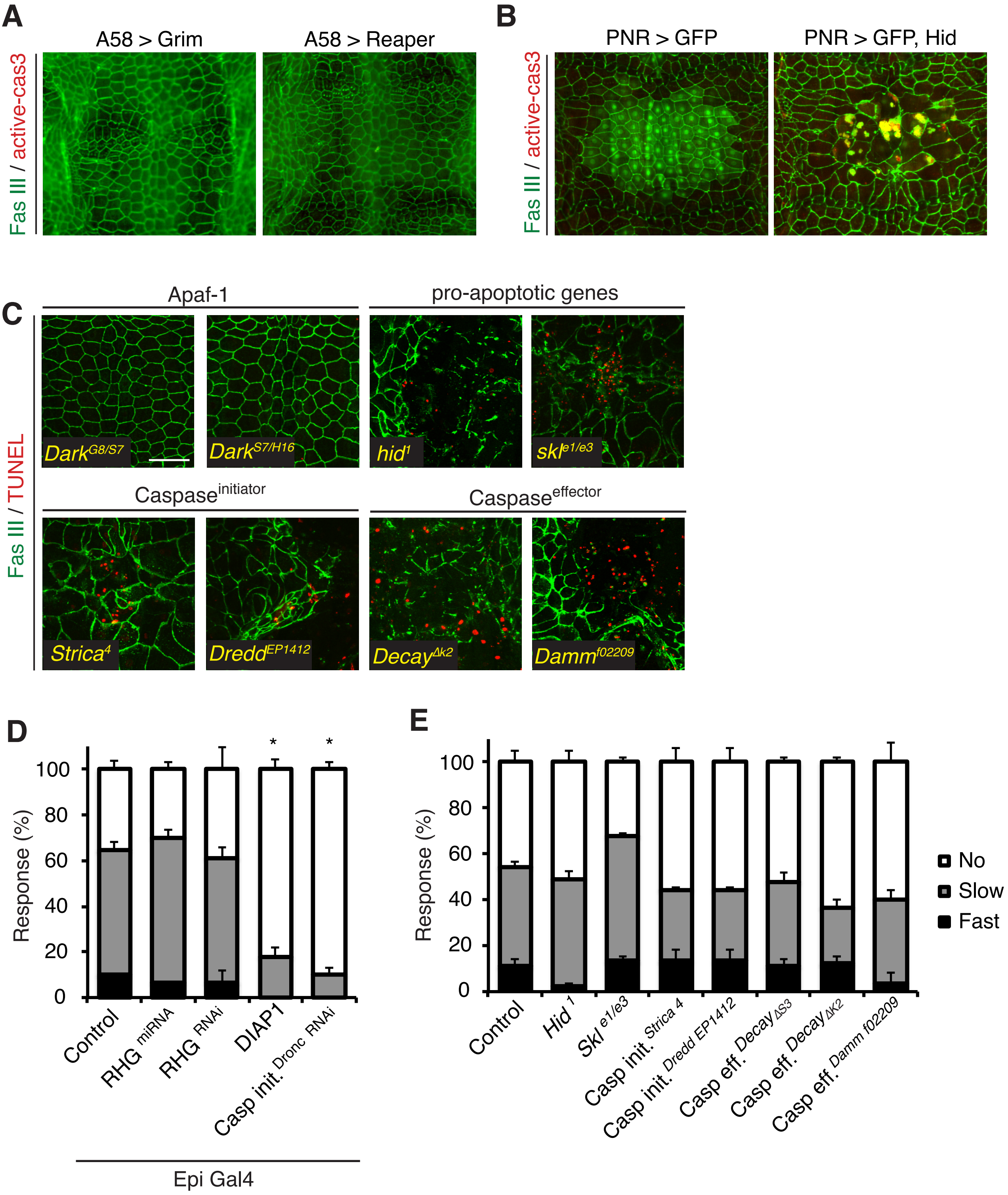
A
A58 > Grim
A58 > Reaper
Fas III / active-cas3
B
PNR > GFP
PNR > GFP, Hid
Fas III / active-cas3
C
Apaf-1
pro-apoptotic genes
Fas III / TUNEL
DarkG8/S7
DarkS7/H16
hid1
skle1/e3
Caspaseinitiator
Caspaseeffector
Strica4
DreddEP1412
DecayΔk2
Dammf02209
D
Response (%)
100
80
60
40
20
0
*
*
Control
RHG miRNA
RHG RNAi
DIAP1
Casp init. Dronc RNAi
Epi Gal4
E
Response (%)
100
80
60
40
20
0
Control
Hid 1
Skl e1/e3
Casp init. Strica 4
Casp init. Dredd EP1412
Casp eff. Decay ΔS3
Casp eff. Decay ΔK2
Casp eff. Damm f02209
No
Slow
Fast

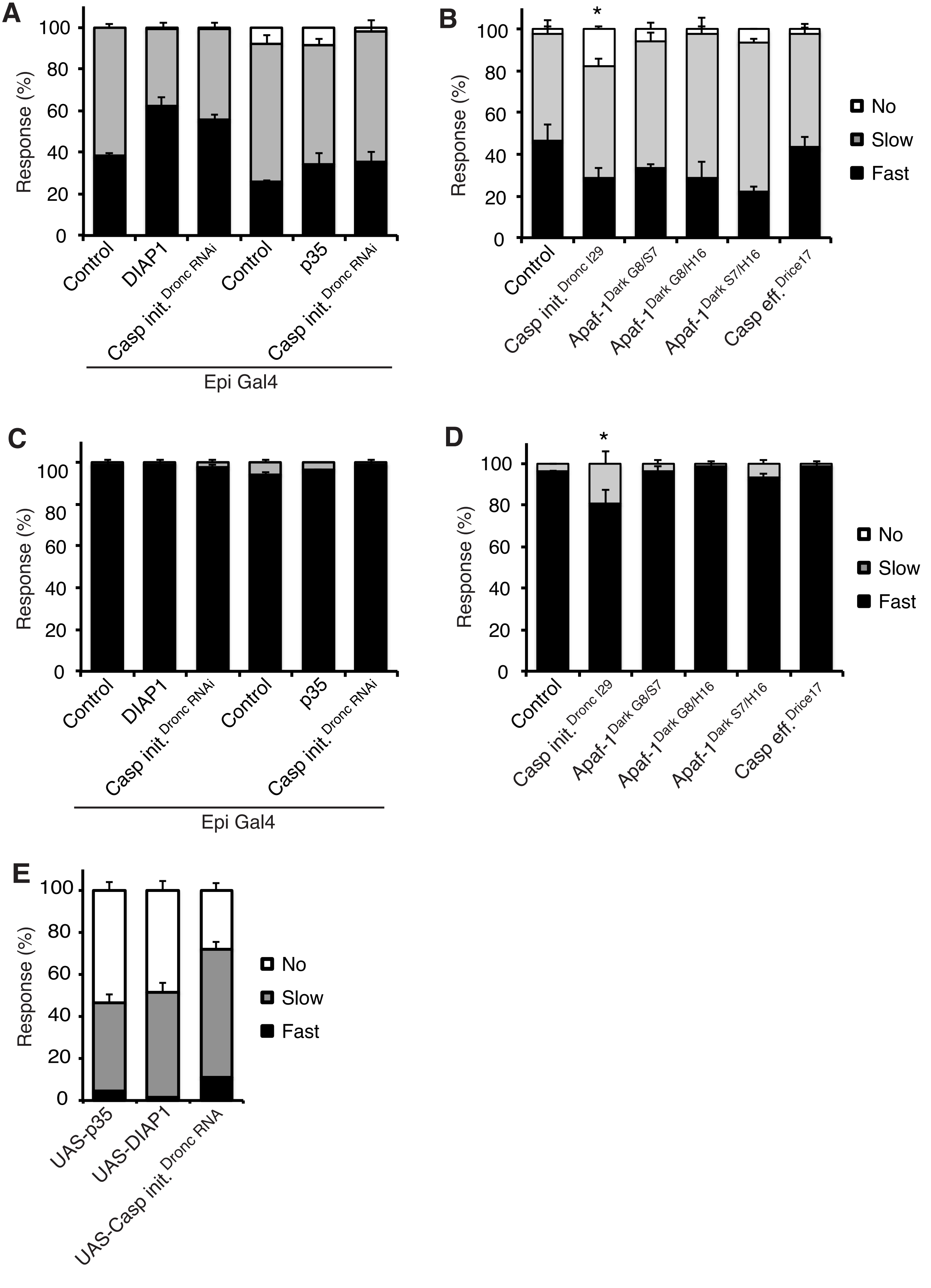
A
B
C
D
E
Response (%)
0
20
40
60
80
100
Control
DIAP1
Casp init. Dronc RNAi
p35
Epi Gal4
*
Casp init. Dronc I29
Apaf-1 Dark G8/S7
Apaf-1 Dark G8/H16
Apaf-1 Dark S7/H16
Casp eff. Drice17
No
Slow
Fast
UAS-p35
UAS-DIAP1
UAS-Casp init. Dronc RNA

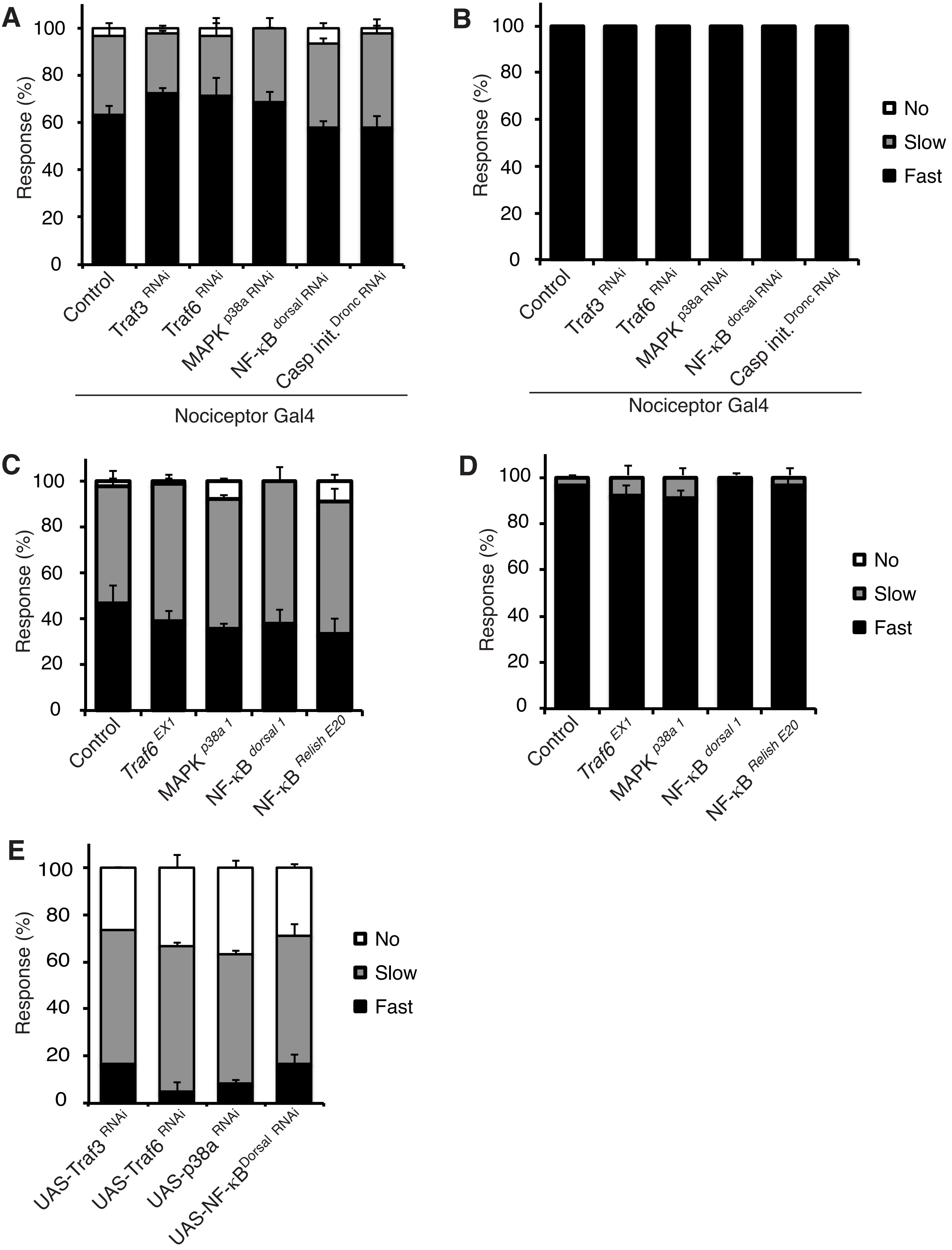
A
B
C
D
E
Response (%)
0
20
40
60
80
100
No
Slow
Fast
Control
Traf3 RNAi
Traf6 RNAi
MAPK p38a RNAi
NF-κB dorsal RNAi
Casp init. Dronc RNAi
Nociceptor Gal4
Traf6 EX1
MAPK p38a 1
NF-κB dorsal 1
NF-κB Relish E20
UAS-Traf3 RNAi
UAS-Traf6 RNAi
UAS-p38a RNAi
UAS-NF-κB Dorsal RNAi

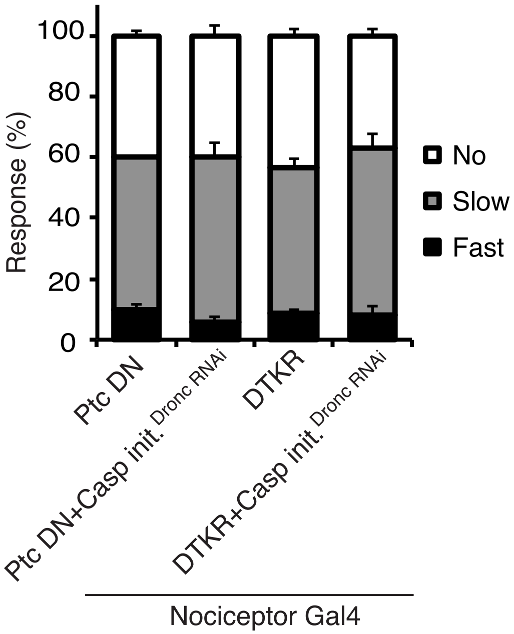

100
80
60
40
20
0
Response (%)
No
Slow
Fast
Ptc DN
Ptc DN+Casp init. Dronc RNAi
DTKR
DTKR+Casp init. Dronc RNAi
Nociceptor Gal4

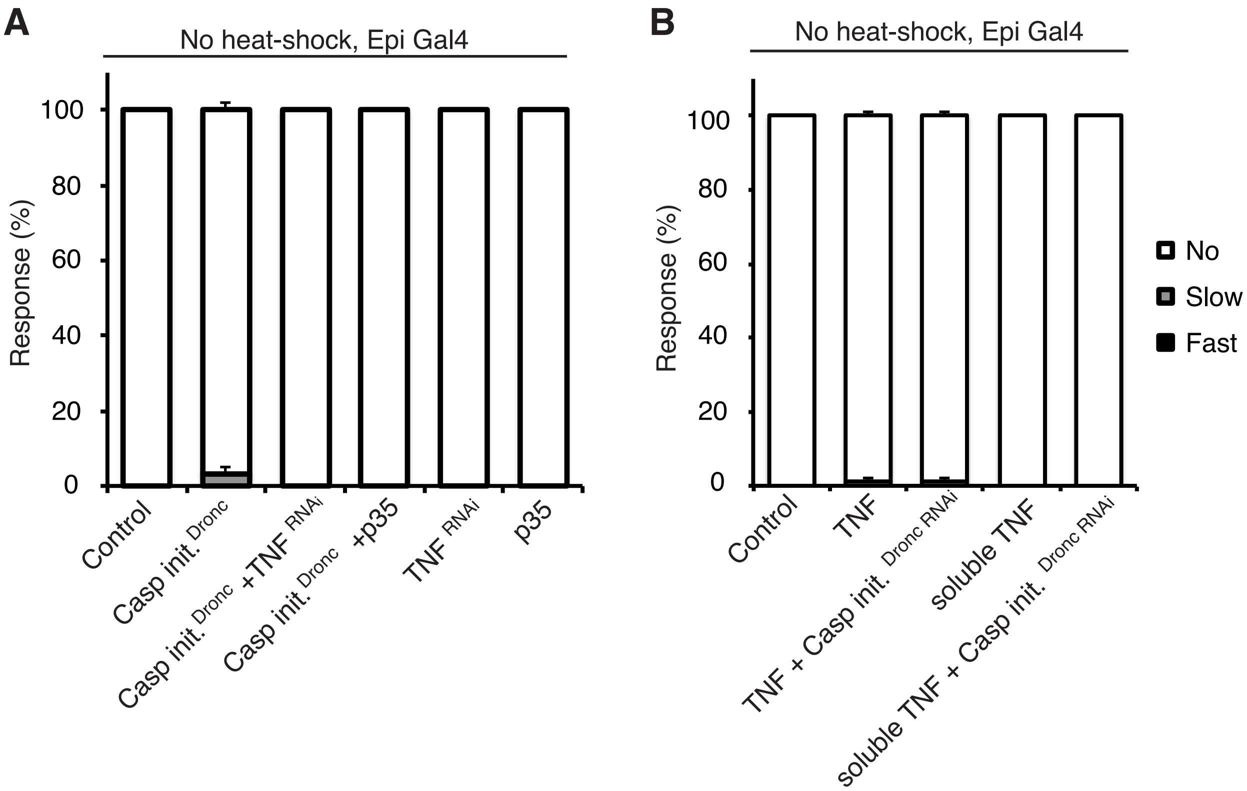

A
No heat-shock, Epi Gal4
Response (%)
100
80
60
40
20
0
Control
Casp init. Dronc
Casp init. Dronc +TNF RNAi
Casp init. Dronc +p35
TNF RNAi
p35
B
No heat-shock, Epi Gal4
Response (%)
100
80
60
40
20
0
Control
TNF
TNF + Casp init. Dronc RNAi
soluble TNF
soluble TNF + Casp init. Dronc RNAi
No
Slow
Fast

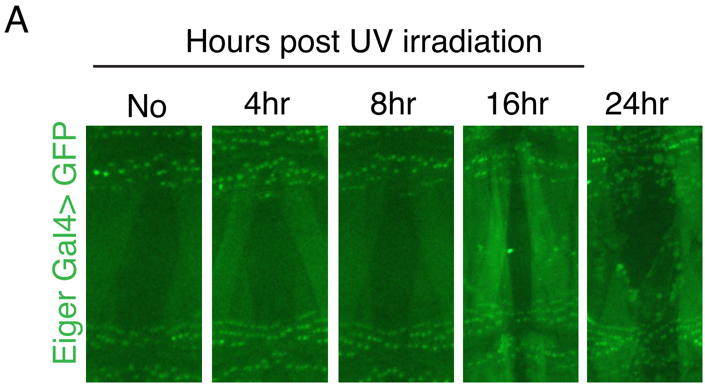
A
Hours post UV irradiation
No
4hr
8hr
16hr
24hr
Eiger Gal4> GFP

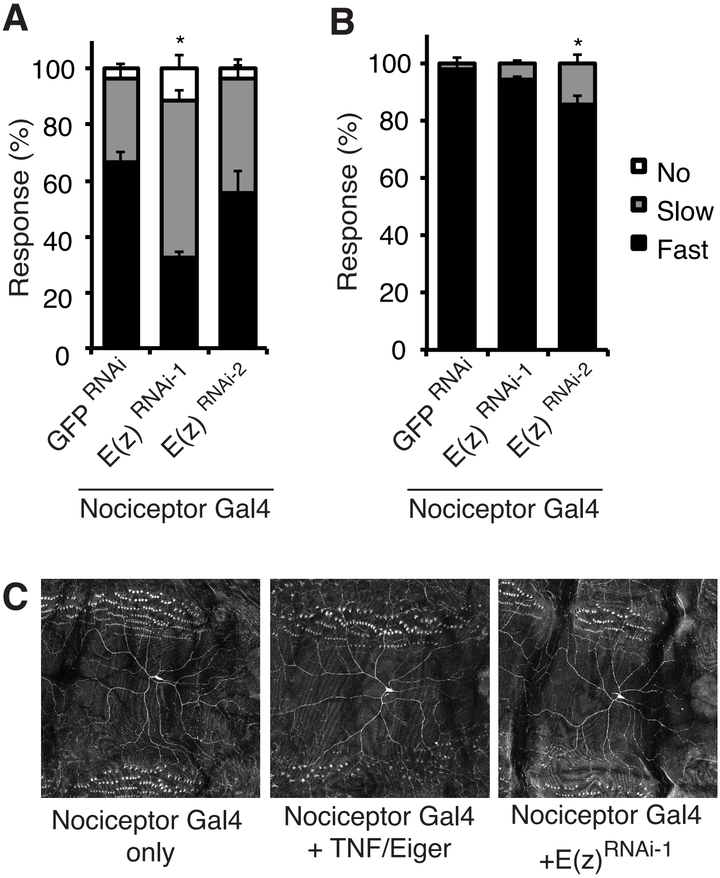

A
B
*
*
100
80
60
40
20
0
Response (%)
No
Slow
Fast
GFP RNAi
E(z) RNAi-1
E(z) RNAi-2
Nociceptor Gal4
Nociceptor Gal4
C
Nociceptor Gal4 only
Nociceptor Gal4 + TNF/Eiger
Nociceptor Gal4 +E(z)RNAi-1
